# Laser-induced choroidal neovascularization: A case report and some reflection on animal models for age-related macular degeneration

**DOI:** 10.1097/MD.0000000000026239

**Published:** 2021-06-11

**Authors:** Caixin Li, Jianqing Li, Xinzhu Chen, Peirong Lu

**Affiliations:** a Department of Ophthalmology, the First Affiliated Hospital of Soochow University; b Department of Ophthalmology, Suzhou EENT Hospital, Suzhou, Jiangsu Province, China.

**Keywords:** age-related macular degeneration, antivascular endothelial growth factor, case report, choroidal neovascularization, laser

## Abstract

**Rationale:**

Laser induced maculopathy includes retinal photoreceptor disruption, macular hole, macular hemorrhage, and rarely choroidal neovascularization (CNV). Here we report a case of laser induced CNV that was treated by intravitreal anti-vascular endothelial growth factor (VEGF) injection and resulted in visual improvement and CNV resolution during 1-year follow up. In addition, the case of laser induced CNV treated with intravitreal anti-VEGF injections are reviewed for the first time in literature.

**Patient concerns:**

A 7-year-old boy presented to our department with blurred vision in his right eye for 2 months. The symptom immediately happened after the boy staring at the laser beam for a few seconds. Examination of ocular fundus with slit lamp showed yellowish lesion in macula in his right eye.

**Diagnoses:**

CNV was confirmed by fundus examinations, including color fundus photograph, spectral domain optical coherence tomography, fluorescein angiography, and spectral domain optical coherence tomography angiography.

**Interventions:**

After the diagnosis of laser induced CNV, intravitreal ranibizumab (LUCENTIS, NOVARTIS) injection was performed.

**Outcomes:**

After 1 injection of intravitreal ranibizumab, the best corrected visual acuity improved from 20/50 to 30/50 and CNV gradually regressed during 1-year follow up.

**Lessons:**

For young patients with laser induced CNV, intravitreal anti-VEGF injections may be helpful in visual improvement and CNV regression. Moreover, age seems to be a significant factor thus we propose that old animals may be more appropriate for laser induced CNV animal models of age-related macular degeneration.

## 1 Introduction

Laser instruments have been applied in many aspects of human activity including industry, military and medicine and entertainment, which has led to more and more accidental eye injuries.^[1]^ As retina and uvea absorb light between 400 and 1400 nm,^[2]^ high-energy laser can cause chorioretinal damage, such as choroidal neovascularization (CNV) in animals^[3]^ and laser induced maculopathy in clinical practice. Laser induced maculopathy has been reported to involve retinal photoreceptor disruption, macular hole, macular hemorrhage and CNV.^[4–6]^ Patients with laser induced CNV typically present with blurred vision, central scotomas, and metamorphopsia.^[7,8]^ The symptoms and the prognosis largely depend on the power and wavelength of the laser and the duration of the exposure.

One of the treatments for laser induced CNV is corticosteroids, which may reduce inflammation caused by the laser, however, the role of steroids remains a debated topic due to variable outcomes.^[7]^ Intravitreal injection of antivascular endothelial growth factor (VEGF) is another treatment because of its antiangiogenetic and anti-inflammatory function.

We herein reported a 7-year-old boy who suffered from accidentally laser induced CNV. After 1 intravitreal injection of ranibizumab (LUCENTIS, NOVARTIS), the CNV gradually regressed during 1-year follow up. Moreover, the cases of laser induced CNV which were treated with intravitreal anti-VEGF injections are reviewed for the first time in literature and that prompts us to think further about the rationality of laser induced CNV as an animal model of neovascular age-related macular degeneration (AMD).

## 2 Case presentation

A 7-year-old Chinese boy presented to our hospital in October 2019 with blurred vision in the right eye (OD) for 2 months. Symptoms occurred immediately after the laser beam of a laser pointer shotting directly into his right eye for a few seconds. The laser pointer is “a class III laser product, wavelength: 650 nm, the maximum output power: less than 100mW” according to the sticker on it. The boy was found to suffer from fundus lesions by a local ophthalmologist and was advised to turn to our department for further diagnosis and treatment. The child was given full-term birth and had no significant past medical history or family history.

**Figure 1:**
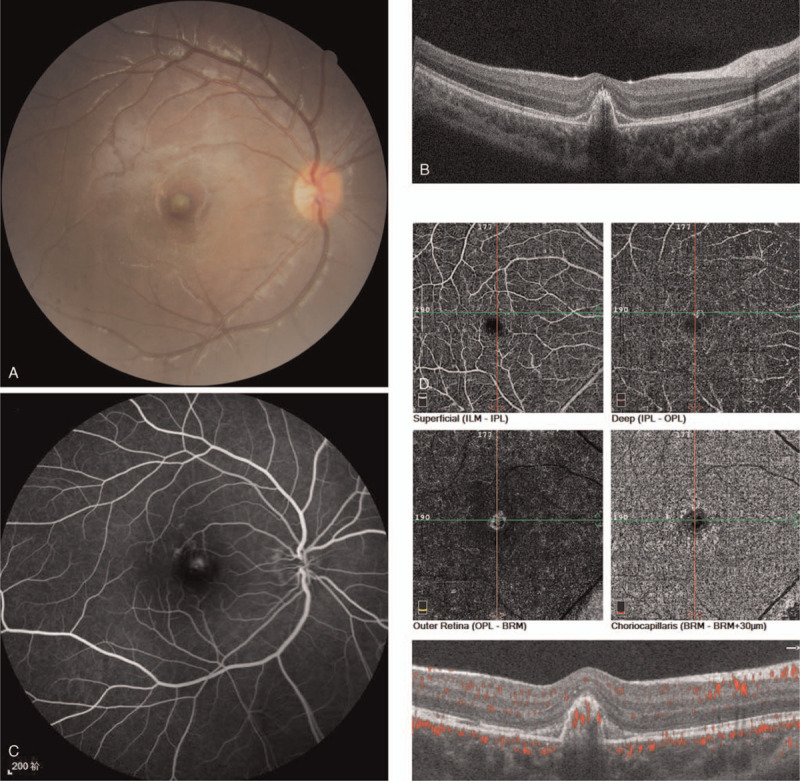
Fundus examinations of the right eye at baseline presentation. A. Color fundus photograph revealed a yellowish-white lesion in the macula. B. Spectral domain optical coherence tomography illustrated disruption of external limiting membrane, ellipsoid zone, interdigitation zone and retinal pigment epithelium, an elevated dome-shaped lesion with an extension to the subretinal space and some subretinal fluid. C. Fluorescein angiography displayed a leaking choroidal neovascular membrane. D. Spectral domain optical coherence tomography angiography revealed the neovascular membrane existing in the level of deep and outer retina and was superonasal to the fovea. Flow deficit in the level of choriocapillaris was found to correspond to the choroidal neovascularization.

On presentation, his best corrected visual acuity (BCVA) was 20/50 OD and 20/20 in the left eye (OS). The intraocular pressures and the anterior segments were normal in both eyes. Funduscopic examination demonstrated a yellowish-white lesion in the macula OD (Fig. 1A). Spectral domain optical coherence tomography (SD-OCT) illustrated disruption of external limiting membrane, ellipsoid zone, interdigitation zone and retinal pigment epithelium, an elevated dome-shaped lesion with an extension to the subretinal space and some subretinal fluid (Fig. 1B). Fluorescein angiography (FA) confirmed a leaking choroidal neovascular membrane (Fig. 1C). Spectral domain optical coherence tomography angiography (SD-OCTA) revealed the neovascular membrane existing in the level of deep and outer retina and was superonasal to the fovea. Flow deficit in the level of choriocapillaris was found to correspond to the choroidal neovascularization (CNV) (Fig. 1D). The OS fundus examination was unremarkable.

**Figure 2:**
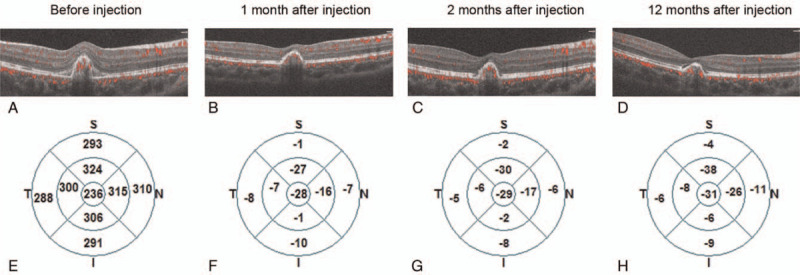
One year follow up after Ranibizumab injection. A-D. Spectral domain optical coherence tomography angiography revealed rapid regression of CNV and complete resolution of subretinal fluid at 1-month follow up, further regression of CNV at 2-month follow up, and the stability of CNV at 12-month follow-up visit. E-H. The change analysis report produced by the optical coherence tomography system displayed the baseline retinal thickness and the changes in retinal thickness relative to the baseline scan at 1-month, 2-month and 12-month follow up.

A diagnosis of laser induced CNV was made. After detailed informed consent from the patient's parents, an intravitreal injection of 0.5 mg/0.05 mL ranibizumab was performed under general anesthesia. At 1-month follow-up visit, the BCVA improved to 20/40 OD. Compared with the SD-OCTA findings before injection (Fig. 2A), rapid regression of CNV and complete resolution of subretinal fluid (Fig. 2B) were observed. Considering the residual CNV, a second intravitreal anti-VEGF injection was advised, yet the parents rejected that because of the child's headache after general anesthesia. At 2-month follow-up visit, the BCVA remained unchanged at 20/40 OD and the neovascular membrane further regressed but still existed (Fig. 2C). We advised an indocyanine green angiography examination for the patient to assess vascular permeability of CNV, however the patients refused to do so because the boy vomited severely during the previous FA examination. At 1-year follow up, the OD BCVA improved to 30/50 and the lesion remained stable (Fig. 2D). From the change analysis report produced by the OCT system, we find a rapid decrease in retinal thickness at the first month after ranibizumab injection and the relatively stability in the following period (Fig. 2E-2H). Thus, a yearly follow up examination was advised to this patient.

## 3 Discussion and conclusions

The incidence of laser induced eye injures has been on a rise because the wide use of laser in many spheres of human activity such as industry,^[9]^ military,^[10]^ medicine,^[11]^ and entertainment.^[12]^ The safety level of laser products is categorized according to the American National Standard Institute and Food and Drug Administration.^[13]^ The laser pointer played with by our patient was labelled as a class III product and was a class IIIb device to be exact (lasers that emit between 5 mW and 500 mW output power), which could not legally be promoted as a laser pointer. Thus, the tragedy immediately happened after the boy staring at the laser beam for a few seconds.

After diagnosis of laser induced CNV, intravitreal ranibizumab was performed. The CNV was observed to regress rapidly in the first month after injection and regress gradually during the 1 year follow up, while the BCVA improved from 20/50 to 30/50. We reviewed the published articles which reported laser induced CNV with treatment of intravitreal anti-VEGF injections. Xu K et al^[8]^ reported a 12-year-old boy and Tofolean IT et al^[14]^ reported a 31-year-old woman whose BCVA improved to 20/20 after only 1 anti-VEGF injection while another 33-year-old patient^[15]^ regained 20/20 after 3 intravitreal injections. Besides, the study groups of Tran K,^[7]^ Forshaw TRJ,^[16]^ Chang CY^[17]^ and Veronese C^[18]^ reported either complete resolution of CNV or visual improvement in young patients (aged between 12 and 26 years old). It appears that anti-VEGF treatment is quite useful for young patients who suffer from laser induced CNV. However, another case reported that a 55-year-old male dermatologist was diagnosed CNV secondary to laser exposure and after 2 intravitreal injections an increase in the size of the CNV with extensive arborization was observed by OCTA.^[10]^ Although there was only 1 case of the elderly patient with laser induced CNV whose anti-VEGF therapeutic effect was poorer than that of young patients, it should be noted that age may be an important factor of anti-VEGF therapeutic effect. CNV is 1 feature in neovascular age-related macular degeneration, which is modeled in animals by laser photocoagulation.^[19]^ However, researchers tend to not take the ages of animals into consideration. Espinosa-Heidmann et al^[20]^ have conducted a research on the impact of age on the severity of neovascularization in a mouse model of laser-induced CNV and they found that age was associated with more severe CNV, defined as larger surface area, greater vascularity, and greater cellularity. Therefore, we suspect that aged animals should be applied to duplicate animal model for AMD, although further studies are required to study the underlying molecular mechanisms on this.

Although the BCVA of our patient restored to 30/50 after 1 anti-VEGF injection, laser exposure left a permanent eye injury on the boy. Apparently, accidental eye injury due to laser exposure has become a serious public health issue which requires more education to prevent this catastrophe.

In summary, we report a 7-year-old boy with laser induced CNV whose BCVA improved from 20/50 to 30/50 1 year after an intravitreal injection of ranibizumab and CNV gradually regressed. In addition, it is the first time in literature to review the cases of laser induced CNV with intravitreal anti-VEGF injections performed and we propose to duplicate CNV animal models for AMD with old animals, which needs further studies to investigate.

## Author contributions

**Conceptualization:** Caixin Li, Peirong Lu.

**Data curation:** Caixin Li, Jianqing Li, Xinzhu Chen.

**Funding acquisition:** Peirong Lu.

**Investigation:** Caixin Li, Jianqing Li, Xinzhu Chen.

**Project administration:** Caixin Li, Peirong Lu.

**Resources:** Peirong Lu.

**Supervision:** Caixin Li, Peirong Lu.

**Visualization:** Caixin Li, Jianqing Li.

**Writing – original draft:** Caixin Li, Jianqing Li.

**Writing – review & editing:** Caixin Li, Jianqing Li, Xinzhu Chen, Peirong Lu.
